# Evaluation and Improvement of the Quality of Surgical Operative Notes in the Department of General Surgery at Dongola Teaching Hospital, Sudan

**DOI:** 10.7759/cureus.70726

**Published:** 2024-10-02

**Authors:** Osama Elhadi Bakheet, Abubakr Muhammed, Ahmed Mohamed, Salih Ahmed Abdalla Ahmed, Abdalrahman Abdalhalim Abdalrahman Ali, Ahmed Salah Ahmed Sayed, Nada Hassan Farah Hassan, Mohammed Eltayeb Elnour Elawad, Mohammed Khalid Mohammedali Abdelrhman, Ayman Adil Eltayeb Abdelnour

**Affiliations:** 1 General Surgery, Dongola Teaching Hospital, Dongola, SDN; 2 General Surgery, University of Gezira, Wad Madani, SDN; 3 Orthopaedics and Trauma, Gezira Centre for Orthopaedic Surgery and Traumatology, Wad Madani, SDN; 4 General Surgery, Wad Madani College of Medical Sciences and Technology, Wad Madani, SDN

**Keywords:** clinical documentation audit, quality improvement (qi), royal college of surgeons, surgery general, surgical notes

## Abstract

Background

Surgical operative notes are essential for patient care and legal documentation. However, inconsistencies in the quality of these notes at Dongola Teaching Hospital, Sudan, highlighted the need for improvement. In line with guidelines from the Royal College of Surgeons of England (RCSEng), this study aimed to enhance the documentation practices in the hospital by implementing a standardized format.

Methods

A retrospective audit was conducted over three months at the General Surgery Department of Dongola Training Hospital. In the first audit cycle, 81 surgical notes were assessed, revealing significant deficiencies in adherence to RCSEng standards. An intervention was introduced, including a standardized template, training for surgeons, and widespread dissemination of the new format. A second audit cycle followed to assess improvements.

Results

In the first audit cycle, adherence to documentation standards was 50.3%, with missing or incomplete information in key areas. After the intervention, adherence improved to 71.9%. Notable improvements included documentation of extra procedures (18% to 100%), prosthesis details (0% to 100%), and antibiotic prophylaxis (71% to 97%). However, a slight decline was observed in postoperative care instructions, dropping from 100% to 90%.

Conclusion

The introduction of a standardized template and training significantly improved the quality of surgical documentation. Continuous efforts are necessary to maintain these improvements, particularly in areas where adherence remains suboptimal, such as postoperative care instructions.

## Introduction

Surgical operative notes are essential in patient records, acting as legal documentation and a vital communication tool within multidisciplinary teams. The Good Surgical Practice guidelines of the Royal College of Surgeons of England (RCSEng) underscore the need for precise, thorough, and timely documentation of surgical procedures to ensure both high-quality patient care and medicolegal protection [1]. These notes offer a comprehensive summary of the surgery performed, support postoperative management, and facilitate continuity of care among different healthcare providers. They are also crucial for clinical audits, quality improvement initiatives, and educational purposes, enhancing patient safety and professional growth [2].

The precision of surgical operative notes is crucial for effective postoperative care and serves as a key medicolegal record [3]. The quality of these records affects medical documentation and the continuity of information essential for patient care. Besides their medical significance, the quality of operative notes carries economic and legal implications, making accurate documentation vital for both audits and enhancing patient care delivery [4]. As per RCSEng guidelines, operative notes should be completed promptly following the procedure to ensure no critical details are missed and to maintain a clear recollection of the surgery. These notes must be legibly and systematically written or dictated, including information on the procedure type, the surgeon’s name, assistant details, surgical techniques, and any complications or deviations from the plan [1,4-6].

Often, junior surgical team members write notes in a rushed manner, leading to incomplete records and the use of unclear abbreviations [6]. This incomplete or inaccurate documentation can lead to serious issues such as delayed or denied reimbursements, particularly when notes are dictated by surgical residents. Additionally, reports authored by residents are frequently incomplete or inaccurate, adversely affecting patient care and hospital financial outcomes [3]. In many hospitals in Sudan, surgeons tend to use blank paper for writing operative notes, which results in inconsistent and incomplete records. The lack of standardized templates can lead to omissions that undermine patient care and legal validity. Implementing structured templates, as advised by the RCSEng, could greatly enhance the quality and uniformity of surgical documentation in these environments [7].

## Materials and methods

This was a retrospective observational audit conducted in the General Surgery Department at Dongola Teaching Hospital, Sudan, over a three-month period from August to October 2023. The primary objective was to evaluate and improve the quality of operative note documentation for all surgeries performed during this period. The study was approved by the Institutional Ethics Committee at Dongola Teaching Hospital (approval number: number 2023/N12). To ensure privacy and confidentiality, all personal and clinical information was anonymized before analysis.

The RCSEng outlines key parameters for surgical operative notes to ensure comprehensive documentation and patient safety. These notes should include the patient's details (name, hospital number, and date of birth), the date and time of the surgery, the names of the operating surgeon and assistants, and the procedure performed. It should also detail the type of anesthesia used and a clear description of the surgical procedure, including findings and any complications encountered. Additionally, the operative note must document the surgical techniques employed, the use of any devices or implants, the closure method, and postoperative instructions, including the need for follow-up care. These parameters ensure that the notes are not only clinically useful but also meet medico-legal standards for thoroughness and accuracy [1].

Pre-intervention phase

The audit began with a detailed review of surgical notes. A total of 81 records were randomly chosen from the department’s archive utilizing a simple randomization technique. This analysis exposed several issues with the documentation, such as incomplete and unstructured operative notes, emphasizing the need for corrective action.

Root cause examination 

A fundamental cause examination was performed to ascertain the elements leading to the inadequacies in the record-keeping of surgical procedures. the key issues identified included the absence of standardized templates for operative notes and insufficient awareness among surgeons about the importance of comprehensive documentation. Additionally, the lack of clear guidelines for documenting surgical procedures contributed to missing essential details such as patient demographics, surgical findings, and postoperative instructions.

Intervention phase

To address the identified gaps, a comprehensive intervention was implemented, which included three primary components: (i) Creation of a standardized operation note format: The previously used unstructured format was replaced with a standardized template based on the Royal College of Surgeons of England's guidelines; (ii) Dissemination of the new template: The standardized template was circulated throughout all surgical departments to achieve consistency in operation note documentation; (iii) Instruction and training: Surgeons and surgical staff received training on the significance of comprehensive and standardized documentation. Training methods encompassed a Microsoft PowerPoint (Microsoft Corporation, Redmond, Washington, United States) presentation at departmental meetings, targeted group discussions, and one-on-one sessions to emphasize the correct use of the new template

Post-intervention phase

Following a one-month period of intervention, the subsequent phase of the audit, which also spanned one month, commenced. Throughout this phase, every surgical operation record from Dongola Teaching Hospital was examined and evaluated to measure the effects of the intervention. The objective was to appraise advancements in the documentation procedure and compliance with RCSEng standards.

Evaluation

Five authors independently evaluated the surgical operative notes in accordance with the RCSEng guidelines. In instances where discrepancies arose among their assessments, a third party who was blinded to the initial evaluations was consulted to resolve the conflicts and ensure the accuracy and reliability of the audit findings. The third party applied a consensus-driven approach, carefully reviewing each note in light of the established criteria to ensure consistency.

Data analysis

Information from both audit phases was examined using Microsoft Excel 2016 (Microsoft Corporation).

## Results

The results of a thorough assessment of compliance with Good Surgical Practice guidelines carried out across two cycles are detailed in Table 1, highlighting the improvements achieved after the implementation of revised documentation practices. The study reviewed a total of 81 surgical records, with 32 records from the first cycle and 49 records from the second cycle. In the first cycle, adherence to documentation standards was approximately 50.3%. This included variances in documenting key elements such as operative procedures and complications. By the second cycle, adherence improved to about 71.9%, reflecting enhanced compliance with documentation criteria. These findings, summarized in Table 1, underscore the effectiveness of the implemented changes in improving surgical documentation and highlight areas for further refinement.

**Table 1:** Documentation in the first and second cycles, along with the corresponding percentage improvement.

Documentation Criteria	First Cycle (N=32), n (%)	Second Cycle (N=49), n ( %)	Improvement (%)
Date	18 (56%)	28 (67%)	10%
Time	11 (34%)	21 (50%)	16%
Elective/Emergency	1 (3%)	16 (38%)	35%
Name of operating surgeon	26 (81%)	38 (90%)	9%
Name of assistant	22 (69%)	36 (86%)	17%
Name of anesthesia personnel	20 (63%)	27 (64%)	2%
Operative procedure carried out	21 (66%)	38 (90%)	25%
Incision	17 (53%)	31 (74%)	21%
Operative diagnosis	13 (41%)	35 (83%)	43%
Operative findings	15 (47%)	35 (83%)	36%
Any problems/Complications	8 (25%)	27 (64%)	39%
Details of closure technique	19 (59%)	31 (74%)	14%
Anticipated blood loss	0 (0%)	31 (74%)	74%
Postoperative care instruction	32 (100%)	38 (90%)	-10%
Signature	6 (17%)	15 (36%)	17%

Table 2 illustrates items of the surgical operative notes that are specific to certain surgical procedures. In addition to the overall improvements, it is essential to recognize that not all documentation items were applicable to every surgery. For instance, deep vein thrombosis (DVT) prophylaxis documentation was not relevant to the surgeries reviewed in either cycle. However, for items where applicability did apply, significant progress was noted. Notable improvements were observed in several areas: documentation of extra procedures surged by 82%, prosthesis serial numbers were consistently recorded (100% adherence), and antibiotic prophylaxis documentation increased by 26%. However, there was a slight decrease in the documentation of postoperative care instructions, from 100% to 90%. The documentation of extra procedures, applicable to 11 surgeries, improved dramatically from 18% adherence in the first cycle to 100% in the second cycle. Similarly, the recording of tissue removed or altered, applicable to 29 surgeries, increased from 76% to 87%.

**Table 2:** Filling up of surgical operative note items that are relevant to specific procedures. DVT: deep vein thrombosis

Documentation Criteria	First Cycle (N=32)		Second Cycle (N=49)		Improvement (%)
	Applicable, n	Filled, n (%)	Applicable, n	Filled, n (%)	
Tissue removed added or altered	29	22 (75%)	38	33 (86%)	11%
Extra procedures	11	2 (18%)	5	5 (100%)	82%
Prosthesis used with serial number	3	0 (0%)	2	2 (100%)	100%
Antibiotic prophylaxis	32	23 (71%)	42	41 (97%)	26%
DVT prophylaxis	0	0	0	0	0

In Table 1, the audit shows significant overall improvements in the documentation of surgical operative notes between the first and second cycles. There was a marked increase in the recording of key details, such as the name of the operating surgeon, operative findings, and anticipated blood loss, reflecting better adherence to documentation practices. However, a notable and unusual finding is the slight decline in the documentation of postoperative care instructions, which contrasts with improvements in other areas. Another area of concern is the persistent under-documentation of signatures, which, while improved, remains a significant gap. These findings suggest that while interventions have been effective, some aspects, such as signatures and postoperative instructions, require further attention to ensure comprehensive and consistent documentation.

In Table 2, which focuses on criteria relevant to specific surgeries, the audit reveals dramatic improvements. The documentation of extra procedures, previously under-recorded, saw a substantial increase, as did the recording of prosthesis serial numbers, which was entirely absent in the first cycle. These findings highlight a stronger commitment to capturing critical details in surgical notes. Additionally, the improvement in documenting antibiotic prophylaxis is noteworthy, though there remains a small gap in achieving full compliance. The data reflects that while structured interventions have had a significant positive impact, continued efforts are necessary to ensure sustained adherence to best practices, particularly in cases where full documentation is essential.

## Discussion

This study reveals a notable improvement in adherence to Good Surgical Practice guidelines, with overall compliance increasing from approximately 50.3% in the initial cycle to 71.9% in the second cycle. These enhancements, especially in documenting additional procedures and prosthesis serial numbers, underscore the effectiveness of structured interventions in improving surgical documentation.

Surgical operative note documentation plays a critical role in ensuring high-quality patient care, supporting medical research, and protecting healthcare professionals from legal liabilities. A clinical audit of surgical records at Jordan University Hospital, using the Surgical Tool for Auditing Records (STAR), revealed significant improvements in documentation quality between 2016 and 2021 [7]. Factors such as the implementation of electronic medical records, systematic auditing, and personnel education contributed to these enhancements. Proper documentation ensures efficient information transfer, facilitates patient management, and aligns with clinical standards, ultimately improving patient outcomes and care continuity [7].

A similar study by Mohamed et al. (2024) in Wad Madani, Sudan, found that adherence to documenting critical details such as the names of the surgeon and assistants, procedure specifics, and antibiotic prophylaxis exceeded 90% [8]. However, both studies identified gaps in documenting incision types and the timing of the procedure. The improvement observed in our second audit cycle (71.9%) is consistent with the significant gains reported in the Wad Madani study, highlighting the ongoing need for continuous education and the adoption of structured templates to enhance surgical documentation.

When comparing our results with global studies, it was seen that Singh et al. (2012) in the United Kingdom also found poor initial compliance in documenting elements such as 'emergency/elective procedure' and 'time of operation,' which were also concerns in our audit [9]. Following educational interventions and the use of memory aids, Singh's study achieved a near 100% compliance in four out of five deficient areas. While our study demonstrated considerable improvements, it did not reach such high compliance levels, suggesting that additional strategies, such as checklist reminders, could further enhance outcomes in our setting.

Similarly, Mulwafu (2018) in Malawi reported difficulties in documenting the time of operation, urgency, and estimated blood loss, with some variables showing initial compliance rates as low as 0% [10]. After implementing targeted interventions, compliance significantly improved, with most areas achieving over 60%. These results align with our study, which also showed substantial progress in documenting complications and additional procedures but encountered challenges in improving the completeness of postoperative care instructions and maintaining legibility, issues also noted in Mulwafu's study.

In Nigeria, Babalola et al. (2016) found that only 37% of operation notes were appropriately completed, with poor documentation often linked to legibility issues and incomplete entries [11]. This finding is consistent with our observations regarding legibility and postoperative care instructions, emphasizing the need to address these persistent issues that affect documentation quality across various regions.

Similarly, Rogers (2008) in South Africa observed that while consultants were more likely to document surgical actions accurately, they frequently overlooked postoperative orders and wound closure methods [12]. Although our study did not differentiate between trainees and consultants, we identified similar gaps in documenting postoperative orders, highlighting the need for consistent and standardized documentation practices among all levels of surgical staff.

In the United Kingdom, Cutting et al. (2014) reported significant improvements in the completeness of operation notes following an initial audit and re-audit, particularly in documenting operative findings, with compliance rising from 94% to 100% [13]. Our study also saw improvements in this area, although challenges remain in documenting postoperative care instructions. The success of structured proformas in Cutting’s study suggests that their implementation could help address these gaps in our setting.

Javid et al. (2020) in India observed marked improvements in documentation quality after a closed-loop audit, with compliance for most variables exceeding 96% in the second cycle [14]. While our study made substantial progress, particularly in documenting blood loss and complications, we did not achieve the same high compliance levels. The introduction of electronic health records, as used in Javid’s study, may have contributed to higher compliance and could be a valuable tool in our context.

Hassan et al. (2023) in Egypt demonstrated that regular audits and feedback led to high compliance rates, particularly in operative record documentation, improving from 86.15% to 95.6% following interventions [15]. Although our study showed similar improvements, we did not reach such high levels of compliance. Hassan’s study emphasizes the importance of ongoing audits and continuous feedback, which could be integrated into our system to drive further enhancements.

In our opinion, the decline in the documentation of postoperative care instructions in this study, despite interventions, stems from ingrained practices among Sudanese doctors who traditionally document these instructions on separate papers rather than within surgical notes. This habit creates a disconnect that can lead to incomplete patient care. To address this issue, it is essential to emphasize the importance of including postoperative instructions directly in the surgical notes. Strategies such as focus group discussions can facilitate understanding and ownership of the change among healthcare professionals, while educational posters can reinforce best practices. Regular training sessions and feedback mechanisms can also help instil the new practice, ensuring comprehensive documentation that ultimately enhances patient outcomes.

Overall, our study’s findings align with global trends where structured audits, education, and standardized templates consistently lead to better documentation quality. While notable progress was made, especially in documenting additional procedures and prosthesis serial numbers, there remains room for improvement in maintaining legibility and ensuring comprehensive postoperative care instructions. These challenges were similarly observed in multiple studies, indicating they are common barriers to achieving full compliance. Future efforts should focus on sustaining the improvements seen in our study, incorporating continuous audits, and potentially adopting digital systems to enhance overall compliance.

It is crucial to recognize the limitations of our study. The relatively small sample size could impact the broader applicability of our results. Moreover, since the study was carried out at only one centre, the findings may not be directly applicable to other institutions.

## Conclusions

This study highlights the impact of targeted interventions in improving adherence to the Good Surgical Practice guidelines of RCSEng. By enhancing structured documentation practices, there was a notable increase in overall compliance, particularly in documenting additional procedures, prosthesis details, and antibiotic prophylaxis. While progress was achieved, challenges remain, particularly in areas such as postoperative care instructions. These findings emphasize the value of ongoing efforts to refine documentation practices and the importance of continuous monitoring to address remaining gaps. Moving forward, it will be essential to maintain and build upon these improvements to ensure thorough and effective surgical documentation across all relevant aspects.

.

## Figures and Tables

**Figure 1:**
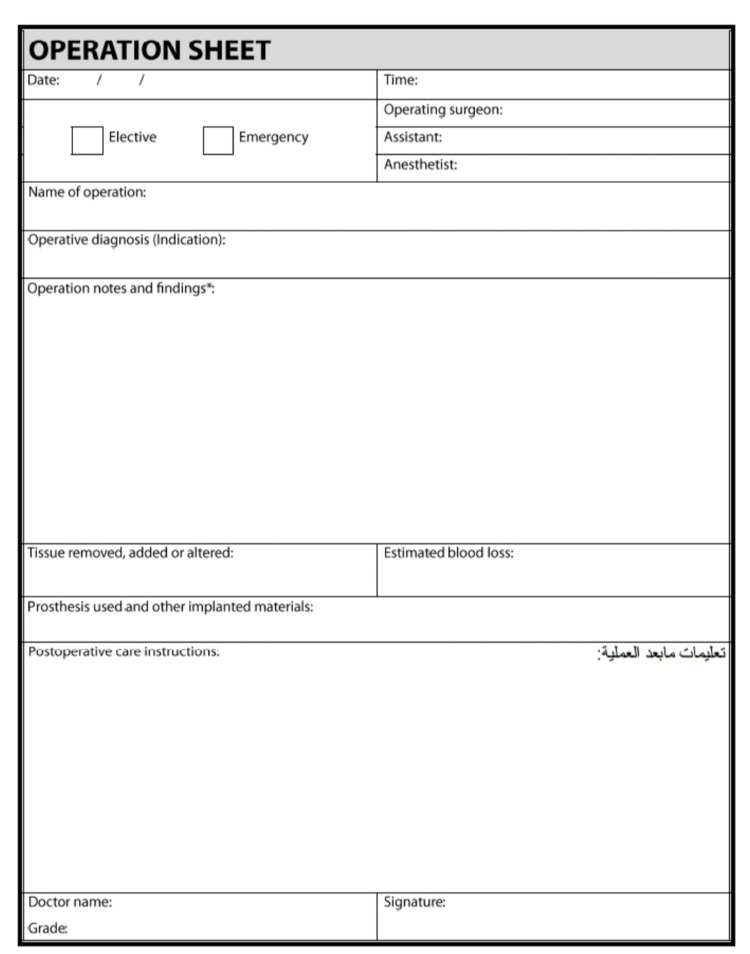
New Surgical Operative Note
